# Genetic Diversity, Runs of Homozygosity, and Selection Signatures in Native Japanese Chickens: Insights from Single-Nucleotide Polymorphisms

**DOI:** 10.3390/ani14223341

**Published:** 2024-11-20

**Authors:** Vanessa V. Velasco, Masaoki Tsudzuki, Norikazu Hashimoto, Naoki Goto, Akira Ishikawa

**Affiliations:** 1 Laboratory of Animal Genetics and Breeding, Graduate School of Bioagricultural Sciences, Nagoya University, Nagoya 464-8601, Japan; t.pom5010@gmail.com; 2 Laboratory of Animal Breeding and Genetics, Graduate School of Integrated Sciences for Life, Hiroshima University, Higashi-Hiroshima 739-8525, Japan; tsudzuki@hiroshima-u.ac.jp; 3 Laboratory of Poultry, Livestock Experiment Station, Hidaka-Gun, Wakayama 644-1111, Japan; hashimoto_n0027@pref.wakayama.lg.jp; 4 Hendrix Genetics BU Layers, 5831 CK Boxmeer, The Netherlands; naoki.goto@hendrix-genetics.com

**Keywords:** genetic diversity, population structure, runs of homozygosity, selection signatures, single-nucleotide polymorphisms, native Japanese chickens, restriction-site-associated DNA sequencing

## Abstract

Understanding genetic diversity, runs of homozygosity (ROH), and selection signatures is critical for the conservation and breeding management of native Japanese chickens. In this study, genetic diversity, ROH, and selection signatures in 11 populations of seven native Japanese breeds and three foreign breeds with different genetic and behavioral backgrounds were investigated using 11,493 SNPs identified through restriction-site-associated DNA sequencing. The Oh-Shamo (OSM), an aggressive cockfighting breed, exhibited the highest genetic diversity. Six native Japanese breeds, Ingie (IG), Ryujin-Jidori (RYU), Tosa-Jidori (TJI), Tosa-Kukin (TKU), Nagoya (NAG), and Ukkokei (UK), showed intermediate levels of genetic diversity and ROH. Population analyses grouped the 11 populations into four distinct clusters: (1) five populations comprising three foreign breeds (the Fayoumi inbred line, closed-colony G line of White Leghorn, commercial T line of WL, and White Plymouth Rock) and the IG Japanese breed; (2) OSM and two old Jidori breeds (RYU and TJI); (3) TKU and UK; and (4) the meat-type Nagoya breed. ROH and Fst analyses identified seven SNPs on chromosomes 13, 17, 20, 24, and 26, five of which were candidate genetic variants for fear-related behavior. These findings provide insights into genetic diversity and conserved genomic segments valuable for breeding and conservation in Japanese chicken breeds.

## Simple Summary

This study assessed genetic diversity, runs of homozygosity (ROH), and selection signatures in 11 populations of seven native Japanese chicken breeds and three foreign chicken breeds using 11,493 single nucleotide polymorphisms (SNPs) from restriction-site-associated DNA sequencing. Six Japanese chicken populations showed moderate diversity, with one breed exhibiting higher genetic diversity. Population analyses grouped the 11 populations into four clusters, revealing generally lower ROH values in Japanese populations than in foreign populations. ROH and Fst analyses identified seven SNPs within the selection signatures, five of which were candidate genetic variants for fear-related behavior.

## 1. Introduction

The poultry industry plays a vital role in agriculture as a key provider of eggs and meat. In Japan, commercial Jidori chickens, known as JAS Jidori, are utility chickens certified under the Japanese Agricultural Standards (JAS). These chickens are mostly obtained by crossbreeding native Japanese chickens with foreign chickens. JAS Jidori chickens are known for their flavorful and high-quality meat, which commands a higher price than general broiler meat. JAS has established qualification standards for Jidori meat, including the requirement that the bloodlines of these chickens must be verified to be 50% or more from native Japanese breeds [1]. By maintaining the purity of these bloodlines, the unique characteristics of native Japanese breeds are maintained, guaranteeing the economic advantage of JAS Jidori chickens. These strict standards guarantee the distinctive taste and quality of JAS Jidori chickens, thus meeting consumer expectations and supporting JAS Jidori’s premium market position.

There are about 45 known native Japanese breeds, 17 of which are designated National Natural Treasures [2]. According to previous literature, certain native Japanese chicken breeds, such as Ryujin-Jidori (RYU) and Tosa-Kukin (TKU), are currently maintained in small numbers [3], whereas Ukkokei (UK), known as Japanese silkie fowl, have remained popular and are bred across the country of Japan [3]. Conservation efforts for bloodlines of native Japanese breeds require adequate measurement of diversities and inbreeding coefficients. Inbreeding coefficients provide valuable data about population viability and genetic diversity, aiding in the decision-making process of conservation strategies. The assessment of inbreeding coefficients is accurately measured in runs of homozygosity (ROH) [4]. ROH are consecutive genomic regions that are identical by descent. A higher number and longer segments of ROH correlate with a higher genomic inbreeding coefficient [5]. In a previous study, an assessment of ROH counts and segments was used to assess the genetic variability of various breeds using germplasms. This study was able to distinguish between crossbreeds and purebreds using ROH analysis [6]. Furthermore, the examination of ROH segments could provide valuable insight into the selection pressures acting on native Japanese breeds and help identify conserved genomic regions.

Beyond their economic importance, poultry species are indispensable for their cultural and educational contributions. Chickens serve as ideal models in basic sciences [7] and have emerged as valuable animal models for conducting in-depth studies of fear, anxiety, stress, and related disorders in humans [8,9]. The neurocircuitry of fear and anxiety are intertwined, including the stress response [10]. Both fear and anxiety are fundamental emotions that play crucial roles in enhancing an organism’s survival and are therefore genetically conserved at the genetic level [11]. Studies of quantitative trait loci (QTLs) associated with fear using native Japanese chickens demonstrated the importance of exploring different genetic breeds to understand genetic variations for fear and related traits. Since the results of association tests for fear and related behaviors are population- and phenotype-specific, searching for genetic resources leads to identifying QTLs and their candidate genes. Fear behavior has been investigated in seven native Japanese breeds: Ingie (IG), Nagoya (NAG), Oh-Shamo (OSM), RYU, Tosa-Jidori (TJI), TKU, and UK [12,13,14,15,16,17,18,19]. QTLs and candidate genes associated with fearfulness have also been reported [16,17,18,19]. Thus, studying the genetic relationships among these breeds may provide insight into the behavioral tendencies that have previously been reported in native Japanese chickens [12,13,14,15,16,17,18,19].

The genetic relationships and variability in native Japanese chicken breeds and commercial foreign breeds have been thoroughly investigated using numerous microsatellite markers and mitochondrial DNA (mtDNA) polymorphisms [20,21]. However, limited studies have been conducted to assess the ROH of native Japanese chickens. Additionally, common genetic variants that are widely spread in the genome, such as single-nucleotide polymorphisms (SNPs), serve as valuable tools to assess genetic diversity, ROH, and selection signatures. In a previous study, whole-genome sequencing identified about 12 million SNPs that distinguished genetic diversities, ROH, and selection signatures in the Cornish, Plymouth Rock, and Ushanka breeds. This study identified conserved genome segments associated with economic traits of varying importance in chickens, including body weight and reproduction [22]. Furthermore, about sixty-six thousand informative SNPs identified by whole-genome sequencing were used to identify conserved genome segments associated with the specific trait of white earlobe color in mediterranean chickens [23]. In a similar study, about 30 million SNPs were used to identify selection signatures for specific traits of broilers, layers, and native chickens [24]. However, the identification of SNPs through whole-genome sequencing is still challenging due to its cost. An alternative to whole-genome sequencing is restriction-site-associated DNA sequencing (RAD-seq), which is a cost-effective method as it targets reduced representations of SNPs across the genome. RAD-seq is an effective tool in phylogeny analysis to compare divergence between populations, subspecies, and related species [25]. Given the imperative to conserve genetic diversity in chickens while managing costs, it is crucial to prioritize the conservation of breeds that contribute significantly to genetic diversity [4]. Thus, this study aimed to delineate the genetic diversities and inbreeding levels across 11 populations of seven native Japanese chicken breeds and three foreign chicken breeds using SNPs obtained by RAD-seq analysis. This represents the first population genetics study using SNPs in native Japanese chickens.

## 2. Materials and Methods

### 2.1. Ethical Note

All laboratory procedures and the handling of chicks were carried out following the protocols established by the Animal Research Committee of Nagoya University (approval numbers: AGR2019016, A210214, A230037).

### 2.2. Chicken Population

Table 1 presents the 11 populations of chicken breeds used in this study. The OSM, TJI, and UK breeds are considered National Natural Treasures. OSM is known for its aggressiveness as it is bred primarily for cockfighting. TJI and RYU are old breeds of native Jidori that show morphological similarities with their chicken ancestor Red Junglefowl [2], although RYU has a slightly larger body than TJI [3]. UK is known as the Japanese silkie fowl. NAG and TKU are classified as utility fowl, both resulting from crossing native Japanese fowl with the Cochin breed [2]. The White Leghorn G line (WL-G) is a closed-colony line maintained at the Avian Bioscience Center Nagoya University, while the White Leghorn T line (WL-T) is the commercial line Babcock B400 and is available from a private company (Tomaru Co., Ltd., Maebashi, Japan). PNP is a highly inbred line that originated from the Fayoumi breed. White Plymouth Rock (WPR) is a breed utilized for meat production.

### 2.3. RAD-Seq Analysis

Blood samples for all of the chickens used in this study were collected in our previous studies [12,15,16,17]. Genomic DNA was extracted from blood using a DNeasy Blood and Tissue Kit (Qiagen, Tokyo, Japan). The quantity of the genomic DNA was measured with a Qubit 3.0 fluorometer (Thermo Fisher Scientific, Tokyo, Japan). RAD-seq was performed by outsourcing to Bioengineering Lab. Co., Ltd. (Sagamihara, Japan). In library preparation, genomic DNA was digested by two restriction enzymes (EcoRI and MspI) for three hours at 37 °C. Then, DNA fragments were harvested and purified using DNA Clean Beads (MGI Tech Co., Tokyo, Japan). The DNA fragments were ligated with annealed adapters using T4 DNA ligase (Takara Bio Inc., Otsu, Japan) for 16 h, followed by another purification using DNA Clean Beads. The purified library was amplified by PCR. Then, the DNA Clean Beads were used to select the sizes of the DNA library. To ensure the concentration of the generated library, SynergyH1 and the QuantiFluor dsDNA System (Promega, Tokyo, Japan) were used, followed by a quality check using a Bioanalyzer and a High-Sensitivity DNA Kit (Agilent Technologies, Santa Clara, CA, USA). The generated libraries were used in the DNBSEQ-G400 Sequencing performed at 2 × 100 bp. Cutadapt ver. 4.0 [26] was used to remove the adapter sequence, followed by the removal of low-quality sequences with quality scores of less than 20 and paired reads with fewer than 40 bases. High-quality sequences were then mapped to the chicken genome assembly GCF_016699485.2 using Bowtie2 ver. 2.5.0 [27]. The Mpileup and bcftools functions of the Samtools ver.0.1.19 program [28] were used to identify single-nucleotide polymorphisms (SNPs) and insertion variants from the mapping data. The SNPs were then filtered with vcftools [29] using thresholds of a minimum quality of 10, a minimum depth of 4, and the deletion of insertion defects. The resulting RAD-seq data were deposited with the DNA Data Bank of Japan (DDJB) under accession numbers DRX438349–DRX438468.

### 2.4. Genomic Analysis

#### 2.4.1. Diversity

Genomic data containing SNP details were handled and analyzed for post-variant filtering using the dartR program [30]. The variant call format (VCF) file of the SNPs was converted to a genlight object using the vcfR package [31]. Bi-allelic SNP markers located on only autosomes were filtered and used in this study. Post-variant filtering parameters were set to an SNP call rate of 95%, an individual call rate of 80%, and the removal of monomorphic SNPs. Before further analysis, linked SNPs were filtered out using Plink 1.07 software [32] based on pairwise genotypic correlations. The parameter for linkage disequilibrium (LD) screening was set to --indep 50 5 0.2, which means a sliding window of 50 SNPs, with a step size of 5 SNPs, and we pruned any SNPs with an LD measure greater than 0.2.

An analysis of molecular variance (AMOVA) was performed within and among the 11 chicken populations using the Poppr program [33] in R. Before the AMOVA, the data were converted from genlight to genind objects using the adegenet package [34]. A significant threshold was set by performing 1000 permutations using the randtest function in the ade4 package [35]. The calcdiversity function of the SambaR package [36] was used to analyze heterozygosity (He), Dxy, Tajima’s D, and the number of private alleles. He was calculated as follows:

$$He=\left(\frac{n_{H}}{n_{ind}\times n_{snps}}\right)\;÷n_{total}$$

where $n_{H}$ was the number of polymorphic sites per individual *i* within the dataset, $n_{ind}$ was the total number of SNP markers without missing values for individuals *i* within the dataset, $n_{snps}$ was the total number of SNPs in the dataset, and $n_{total}$ was the genome length covered by the dataset. Tajima’s D assessed the occurrence of rare alleles within the populations and was computed as the difference between Pi (π) and Theta (θ). Nucleotide diversity or π was defined as the expected proportion of differences between any two randomly drawn sequences, while θ was defined as the number of differences between two randomly drawn haplotypes within the population to which the individual belongs. Details of the computation are presented elsewhere [36]. A chi-square test for Tajima’s D, π, and θ was conducted using the function of SambaR package [36]. A negative Tajima D implied selection sweep, population growth, weak purifying selection, or weak sampling design, while a positive Tajima’s D indicated balancing selection or population expansion after a bottleneck [37]. The number of private alleles was the number of unique alleles in a single population compared to the other populations.

#### 2.4.2. Population Structure

Population structure was analyzed using structure 2.3.4 [38], using admixture model for 10 independent runs in each cluster (K) of genetic populations, ranging from 2 to 11, following a 10,000-iteration burn-in period and a 10,000-repetition Markov chain Monte Carlo (MCMC). To identify the optimal K, an Evanno test was performed using the pophelper program in R [39]. The clumppExport function of the pophelper program was used to generate files for CLUMPP v.1.1.2b [40] that permute the cluster output by independent runs of structure. Furthermore, principal coordinate analysis (PCoA) was performed using the gl.pcoa function in dartR [30] and the results were plotted using ggplot2 [41].

#### 2.4.3. Relationships

The genetic distances between populations were assessed using the Euclidean and pairwise fixation index (Fst) distances. The Euclidean distances were calculated using the gl.dist.pop function of the dartR package [30]. The pairwise Fst distances were calculated using the stamppFst function with 1000 bootstrap iterations from the StAMPP-1.6.3 package [42]. Phylogenic trees were illustrated using the gl.tree.nj function in dartR [30].

#### 2.4.4. Runs of Homozygosity (ROH)

The ROH per population were analyzed using the PLINK 1.09 package with the --homozyg function [43]. LD decay was identified by plotting the average LD pairwise values against genetic distance per population using ggplot [41]. In the ROH analysis, the size of the sliding window and SNP thresholds were set according to previous studies of ROH in chickens [22,23,24,44]. The size of the sliding window was 50 SNPs across the genome, while the number of heterozygotes and the number of missing calls allowed in a window were set to 1 and 5, respectively. The proportion of homozygous individuals in any given SNP was set to 0.05, and the maximum gap size between consecutive homozygous SNPs was set to 1000 kb. With a total autosome size of about 930.82 Mb, the genomic inbreeding coefficient (F_ROH_) based on the ROH length was calculated using the formula

$$F_{ROH}=\frac{total\;ROH\;lenght}{total\;genome\;size}$$

The percentage incidence of SNPs within the ROH segment was calculated by counting the occurrence of each SNP in an ROH segment and dividing by the number of individuals. ROH islands, representing regions with a high incidence of SNPs across the populations, were identified by selecting the top 1% of SNPs based on ROH incidence.

#### 2.4.5. Selection Signature

SNPs associated with selection signatures were identified as overlapping between the top 5% of SNPs with the highest ROH incidence and the top 1% of SNPs with the lowest *p*-values for the highest fixation index (Fst) from the OutFLANK program analysis [45]. SNP outliers were determined in the OutFLANK program based on the distribution of neutral Fst. Outlier SNPs were identified by a chi-square test that compared the observed Fst values to the expected neutral Fst distribution to calculate the *p*-values. Next, overlapping SNPs between high ROH incidence and high Fst values were annotated to identify associated genes using Ensemble’s *Gallus gallus* genome browser https://asia.ensembl.org/Gallus_gallus (accessed on 5 March 2024). Additionally, QTLs associated with behavioral traits were retrieved from the Animal QTLdb database [46] to identify segments where SNPs associated with selection signatures overlapped with QTLs affecting fear and related behaviors.

## 3. Results

### 3.1. SNP Filtering

A total of 120 chickens from 11 populations were RAD-sequenced to assess genetic diversity and ROH. The raw reads of the RAD-seq data initially identified 145,603 SNPs. After post-variant screening, 119 individuals with 43,735 SNPs were retained, meeting thresholds of a 95% SNP call rate and an 80% individual call rate. Subsequently, to eliminate diversity and ROH measurements that might occur by chance rather than due to reflecting true genetic diversity [36] and inbreeding [32], filtering through the linkage disequilibrium (LD) coefficient (R^2^ < 0.2) resulted in 11,493 SNPs remaining.

### 3.2. Genetic Diversity

The AMOVA results revealed that the molecular variance among the 11 chicken populations was 48.21% and the variance within individuals was 51.47%. However, no significant differences were observed among individuals within each population (Table S1).

Table 2 presents the diversity measurements across the chicken populations. The number of segregating bi-allelic SNPs ranged from 11,100 to 11,384, while the minor allele frequency (MAF) ranged from 0.34 to 0.86. Populations with higher values for segregating sites, private alleles, He, π, and θ were indicative of greater genetic diversity among individuals in the population, with the OSM and WPR populations showing the highest diversity. Although PNP had the highest number of segregating sites or number of loci that show variations, PNP and WL-G showed the lowest diversity in terms of private alleles, He, π, and θ. The rest of the populations showed intermediate values for private alleles, He, π, and θ.

The result of Tajima D’s analysis showed significant differences for the chi-square tests. According to the literature [33], positive Tajima D’s values suggest a high number of intermediate-frequency alleles, potentially due to recent population expansion following bottlenecks or selective sweeps, while negative Tajima D’s values suggest an excess of low-frequency alleles, which may result from population contraction or balancing selection. Positive Tajima D’s values were recorded with NAG, OSM, TKU, UK, WL-T, and WPR, while negative values were recorded with IG, RYU, TJI, PNP, and WL-G.

### 3.3. Population Structure

**Figure 1:**
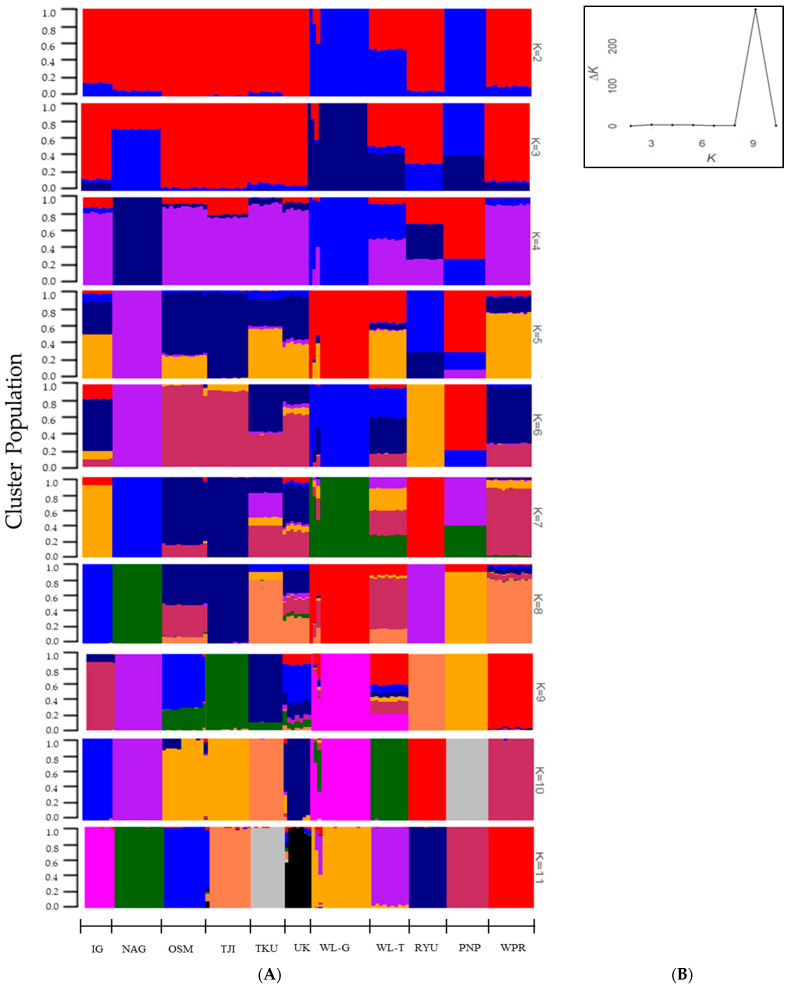
The results of the structure analysis. (**A**) Structure bar plots of the native Japanese and foreign chicken populations (**B**) An Evanno plot showing the ΔK (Delta K) values against the number of clusters (K). Each bar represents an individual chicken genome, and colors represent the number of K. The *x*-axis indicates the population, and the *y*-axis shows the proportion of each cluster within individual genomes.

The population structure based on different numbers of clusters (K = 2 to 11) is presented in Figure 1A. Based on the Evanno test, the optimal K was 9, with the highest value of ΔK (Figure 1B). The proportion of each cluster per individual at K = 9 is illustrated in Figure 1B. Pronounced admixture was observed in five populations of IG, OSM, TKU, UK, and WL-T. Among the Japanese populations, UK shared common SNPs with all populations, while the commercial WL-T population shared SNPs with three foreign chicken populations (WPR, WL-G, and PNP) and three Japanese populations (IG, OSM, and TKU). The OSM population shared SNPs with the TJI and TKU populations, and the IG shared SNPs with TKU. In contrast, the NAG, RYU, and PNP populations were characterized by having different SNPs.

**Figure 2:**
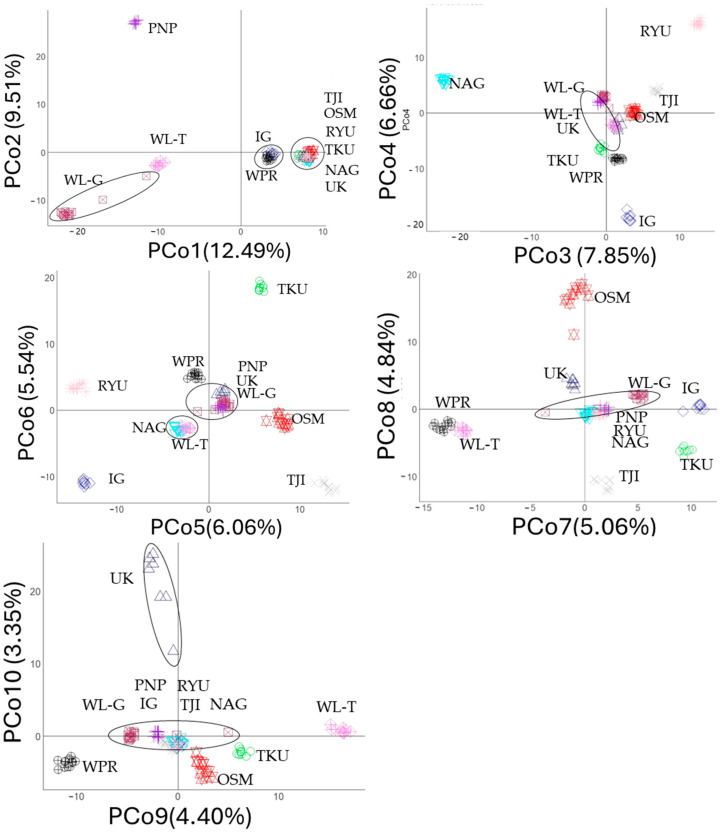
Principal coordinate analysis (PCoA) of SNPs showing population clustering across different principal coordinate (PCo) axes. Eleven populations are represented by unique combination of shapes and colors.

Furthermore, the results of PCoA are shown in Figure 2. The eigenvalues of the first eleven principal coordinate (PCo) axes are presented in Figure S1. Consistent with the optimal clustering (K = 9) identified in the structure analysis, the eigenvalue graph displayed an “elbow” at the ninth principal coordinate (PCo9) axis (Figure S1). This indicated that the first nine PCo axes explain most of the variations in the SNP data. While populations were generally distinct across PCo axes, some clustering between populations was also observed.

On the PCo1 and PCo2 axes, native Japanese populations formed a closer cluster, separate from foreign populations, except for IG. IG clustered more closely with WPR. On PCo3 and PCo4, RYU, NAG, and IG were isolated from the other populations. A similar trend was observed for later coordinates. IG, RYU, TKU, TJI, and OSM were isolated on PCo5 and PCo6. On PCo7 and PCo8, WLG clustered with the PNP, RYU, and NAG populations. Finally, on PCo9 and PCo10, WL-G clustered with PNP, RYU, IG, TJI, and NAG.

### 3.4. Genetic Relationships

**Figure 3:**
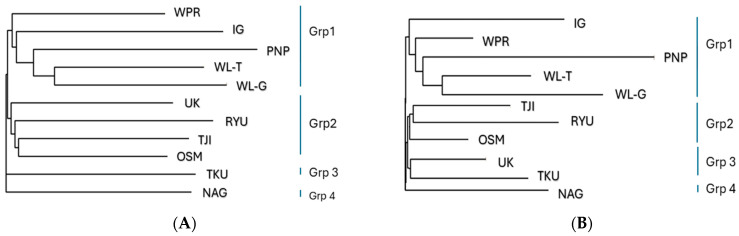
Phylogenetic tree of native Japanese and foreign chicken populations based on (**A**) Euclidean distance and (**B**) pairwise Fst distance.

The genetic relationships based on the Euclidean distances and pairwise Fst distances are visualized in Figure 3. Longer branches indicate greater genetic differentiation. For both the Euclidean and Fst distances, the maximum genetic distance was recorded between the PNP and IG populations, indicating the farthest genetic relationship, while the lowest value of genetic distance was recorded between the OSM and UK populations (Table S2).

Using Euclidean distances, which reflect the similarity of SNPs, the 11 chicken populations were clustered into four groups. Group 1 included the WPR, IG, PNP, WL-T, and WL-G populations; Group 2 included the UK, RYU, TJI, and OSM populations; Group 3 included only the TKU population; and Group 4 included only the NAG population. TKU and NAG were individually isolated and had the lowest genetic similarity to the other groups (Figure 3A).

The Fst distances based on differences in SNP allele frequencies clustered the 11 populations similarly to the groupings observed for the Euclidean distances. Group 1 (WPR, IG, PNP, WL-T, and WL-G) remained unchanged, while Group 2 included RYU, TJI, and OSM, but not UK. In Group 3, UK and TKU were more closely related, while NAG remained separated in Group 4.

### 3.5. Runs of Homozygosity (ROH)

**Figure 4:**
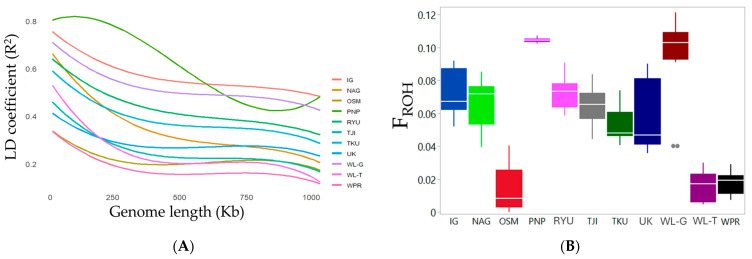

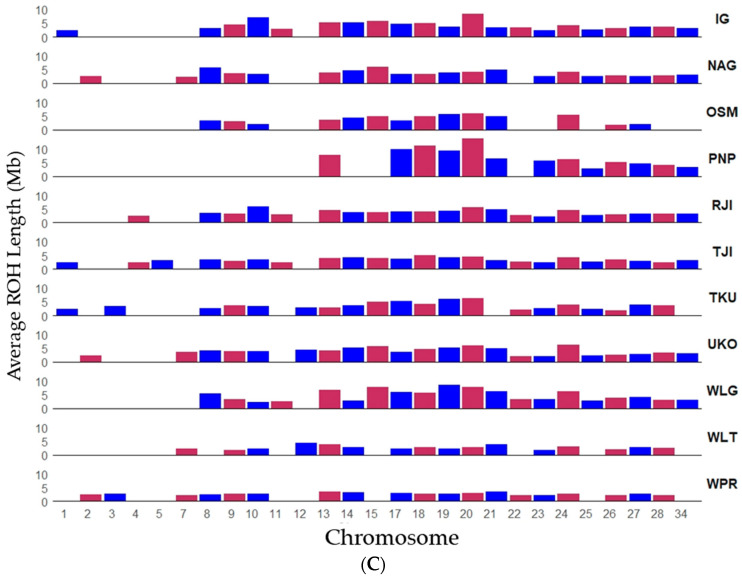
Linkage disequilibrium (LD) decay and runs of homozygosity (ROH) in native Japanese and foreign chicken populations. (**A**) LD decay with increasing physical distance between single-nucleotide polymorphisms (SNPs). (**B**) Average inbreeding coefficients (F_ROH_), with box plot illustrating range and distribution of F_ROH_ among chicken populations. Outlier data points are represented as dots outside the “whiskers” of the boxplot, indicating values that fall beyond 1.5 times the interquartile range (IQR). (**C**) Average sizes of ROH segments across genome for each population. Chromosomes are represented by alternating colors (maroon and blue).

Figure 4A shows the LD decay that distinguishes the 11 populations. Higher LD decay was expected in isolated populations with lower recombination rates, which was consistent with the findings in the PNP inbred line and the WL-G closed-colony line. WPR, WL-T, and OSM had the lowest LD decay, while the remaining chicken populations maintained an LD coefficient (R^2^) value of more than 0.2 for longer chromosomal segments. In this connection, a total of 1502 ROH segments were identified (Table S3). As expected, the F_ROH_ values were highest in PNP and WL-G (Figure 4B), indicating that both PNP and WL-G had longer ROH segments. In PNP, the ROH segments on chromosomes 13 and 17 through 21 were notably longer, while in WL-G, longer ROH segments were found on chromosomes 8, 13, and 15 through 21 (Figure 4C). Details of ROH segments per chromosome are presented in Table S4. In contrast, the lowest F_ROH_ values were observed in OSM, WL-T, and WPR. In OSM, shorter ROH segments were found on fewer chromosomes, while in WL-T and WPR, shorter ROH segments were more widely distributed throughout the genome. In addition, ROH fragments were prominently detected on chromosomes 13; 17 through 20; 24; and 26 to 28, but not on chromosomes 1 through 7; 11 to 12; and 34.

### 3.6. Selection Signature

**Figure 5:**
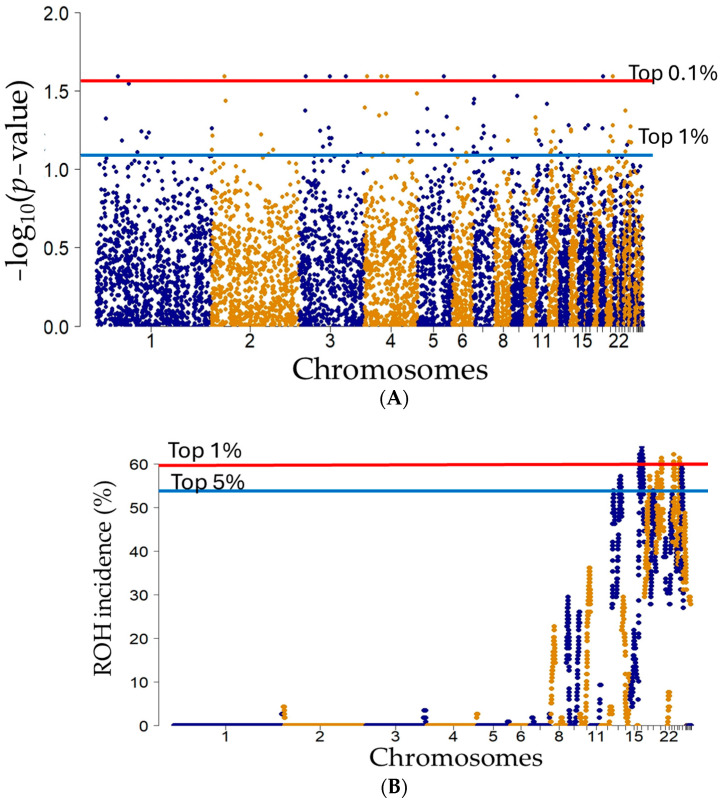
Manhattan plots of (**A**) log10 (*p*-values) of Fst and (**B**) ROH incidence (%) from the OutFLANK program analysis. In (**A**), the red and blue lines indicate the top 0.01% and top 1% thresholds, respectively. In (**B**), ROH incidence indicates the percentage of SNPs within ROH segments across chromosomes. The red and blue lines indicate the top 1% and 5% thresholds, indicating regions of higher ROH concentrations. Each point in the Manhattan and ROH incidence plots represents an individual SNP marker. Chromosomes are represented by alternating colors (light brown and dark blue).

No SNPs showed sufficiently high Fst values to be considered outliers in the OutFLANK program analysis (Table S5). Therefore, SNPs in the top 1% with the highest probability of being outliers in the chi-square test, as indicated by the OutFLANK analysis, were selected to explore potential selection signatures by overlapping with the top 5% of SNPs with the highest ROH incidence. A total of 91 SNPs were identified in the top 1% of SNPs with the lowest Fst *p*-values (Figure 5A and Table S5), and 603 SNPs were selected in the top 5% of ROH incidence (Figure 5B and Table S6). Seven SNPs met both the Fst and ROH incidence thresholds (Table 3).

The seven SNPs in the selection signatures were distributed on chromosomes 13, 17, 20, 24, and 26 (Table 2). These SNPs are genetic variants in the *SHOOM1*, *ZNF618*, *RTFDC1*, *ZFP217*, *LOC107055055*, *KCND3*, and *NGF* genes. Five of the seven SNPs were located on or near QTL regions that have been reported to be associated with fearfulness (tonic immobility duration) and aggressive behaviors (aggressiveness, feather pecking, and receiving feather pecking) in chickens [46].

## 4. Discussion

In this study, 11,493 informative SNPs obtained through RAD-seq analysis were used to determine genetic diversity, genetic relationships, and ROH of seven native Japanese breeds and three foreign breeds. The observed He, Tajima’s D, and Fst distances between populations were lower than previously reported genetic measurements of native Japanese chickens and commercial breeds using microsatellite markers [20,21,47,48]. The differences between the genetic measurements from the SNPs and microsatellites may result from variations in mutation rates, the number of loci, and population sizes. In smaller microsatellite marker sets, high mutation rates can inflate genetic diversity estimates, while increasing the number of SNPs may deflate genetic diversity estimates [49]. Furthermore, small population sizes can affect He values [49]. Previous studies have also shown that SNPs yield lower He values, Fst distances, and ancestry coefficients than microsatellites. Although genetic diversity measures are correlated between microsatellites and SNPs, SNPs provide a more precise assessment of population diversity and structure [49,50,51].

In the present study, the OSM population exhibited the highest genetic diversity, as evidenced by the number of segregating sites, He, π, θ, and Tajima’s D. This finding aligns with a previous study that reported higher genetic diversity in OSM and UK among native Japanese chicken breeds based on mtDNA polymorphisms and microsatellite markers [21]. The genetic diversity of the WL-T and WPR populations was found to be higher than that of native Japanese chicken populations, except for OSM. WL-T is a foreign commercial utility chicken bred by the Tomaru Corporation, a private Japanese company, by crossing four lines of WL to obtain specific traits. WPR is another foreign breed and is maintained by the NLBCH, a Japanese public agency, as the parent stock for commercial broilers. These breeding strategies likely contribute to the higher genetic diversity observed in the WL-T and WPR populations. In contrast, the inbred PNP and closed-colony WL-G lines demonstrated the lowest diversity. These lines have been maintained at Nagoya University since the 1970s [52]. Lastly, the IG, NAG, RYU, TJI, TKU, and UK populations showed lower diversity than two commercial foreign populations (WPR and WL-T) but higher than the inbred PNP and closed-colony WL-G lines. Higher π and θ in NAG, OSM, WL-T, and WPR led to positive Tajima D’s values, indicating higher He, and suggesting balancing selection in these populations. In contrast, negative Tajima D’s values and low He in IG, RYU, TJI, PNP, and WL-G suggest recent selective sweeps. Despite high private allele counts and He in UK, the equal π and θ values indicated that the UK population follows neutral evolution theory. These data reflect demographic history and selection pressures within these chicken populations. Although native Japanese breeds exhibited higher diversity than inbred and closed-colony lines, this finding suggests the need for strategic conservation measures and breeding management to further increase genetic diversity, which is vital for the long-term survival of these breeds.

Through the utilization of 11,493 informative SNPs, the 11 populations were clustered, with the optimal K = 9 determined by structure analysis and PCoA. The population structure inferred from the SNP data provided clearer distinctions between populations compared to a previous study using mtDNA sequencing. In that prior structure analysis, 37 Japanese chicken breeds, including the nine used in the present study, were clustered into two groups using mtDNA sequencing [19]. Generally, SNPs provide higher accuracy in clustering populations [43,44,45]. Notably, the UK population shared common SNPs with all populations in the present study, which might suggest a history of widespread crossbreeding involving UK. According to the literature, UK has been widely raised throughout Japan due to its unique silky feathers and small body size [3]. Furthermore, the foreign chicken WL-T population shared common SNPs with both foreign and Japanese chicken populations. Moreover, OSM shared common SNPs with the TJI and TKU populations. OSM was originally bred for cockfighting and was later used to improve meat quality [2]. TJI is the smallest and oldest Jidori breed, and TKU is crossbred between Cochin-type chickens and native chickens from Kochi Prefecture, Japan. The TKU population also shared SNPs with IG. Lastly, the NAG, RYU, and PNP populations displayed distinct SNP profiles from one another with no admixture. Although NAG was isolated at K = 3 in the structure analysis, PCoA showed close NAG clustering with native Japanese breeds and WL-T. Furthermore, TJI shared common SNPs with RYU up to K = 6 and showed closer clustering with other native Japanese breeds. PNP also shared SNPs with WL-G and WL-T until K = 8 and clustered with WL-G, NAG, UK, and RYU in PCoA. Despite the distinct SNP profiles observed in NAG, RYU, and PNP at optimal k = 9, traces of admixtures were evident in the PCoA and phylogenetic trees based on the Euclidean and Fst distances.

The genetic relationships inferred from the Euclidean and Fst distances revealed closer genetic relationships between specific chicken populations. Specifically, genetic clustering grouped three foreign populations (PNP, WL-G, and WL-T) with the Japanese IG population. This finding is consistent with a previous study in which IG shared common mtDNA haplotypes with foreign breeds, including WL, Barred Plymouth Rock, Rhode Island Red, Araucana, and Brahma [21]. The foreign breeds in this study were primarily subjected to selection pressure for egg and/or meat production, whereas IG was historically maintained as a utility fowl and is now partially preserved as a fancy fowl [3].

The second group included the OSM, UK, RYU, and TJI populations based on the Euclidean distances, but the UK and TKU populations showed closer genetic relationships based on Fst differentiation. Historical evidence suggests that breeding practices differ among breeds in this cluster. For example, OSM is part of the cockfighting breed family [2,53]. TJI is the smallest Jidori breed [2]. Although TJI and RYU both belong to old Jidori breeds, they are classified into different clades based on whole mtDNA D-loop sequences and microsatellite markers [20,21,54]. Both UK and TKU have a meat-type body shape [20], but NAG, despite also having a meat-type body shape, showed a more distant genetic relationship from the other breeds. In recent years, NAG chickens have been selectively bred for egg or meat production [2,3].

Interestingly, the clustering of chicken populations in this study corresponded with distinct behavioral characteristics. PNP, WL-G, and IG exhibited higher activity levels in an open field (OF) test, characterized by faster and more extensive movement as well as bustling behavior [12,15]. In contrast, Group 2, consisting of OSM, UK, RYU, and TJI, differed in their susceptibility to fearfulness. OSM was more aggressive in a handling test [15] and showed reduced fearfulness in both tonic mobility and OF tests [10]. TJI and UK were reported to be sensitive to fearfulness in OF tests [12], while RYU exhibited bustling behavior in the handling test [15]. TKU and NAG, classified into different groups using Euclidean distances, are known to be docile [15] and tame, respectively [3]. Overall, the genetic relationships among these chicken populations may reflect their breeding purposes (egg type, meat type, and fancy fowl) and behavioral characteristics.

The evaluation of F_ROH_ values and ROH segment sizes clearly distinguished inbreeding levels among the populations. In particular, the F_ROH_ values indicated that the inbreeding levels of IG, NAG, RYU, TJI, and UK were higher than those of inbred and close-colony lines, but lower than those of the commercial breeds WL-T and WPR. This suggests the need for intervention to increase heterozygosity and genetic diversity in the five breeds IG, NAG, RYU, TJI, and UK. Furthermore, OSM exhibited the lowest F_ROH_ value and had fewer and shorter ROH segments, making it an ideal breed for further genomic exploration.

This study identified seven SNPs in selection signatures. Five of these SNPs are located within or near QTL regions linked to fearfulness and aggressiveness. Among the identified candidate genes for selection signatures, the *NGF* gene is a promising candidate gene for chicken behavior, as *NGF* encodes a protein that enhances cholinergic neuron function in the cholinergic basal forebrain [55] and regulates synaptic plasticity [56]. Additionally, *NGF* affects anxiety behavior and conditioned fear in mice [57]. *KCND3* has also been reported to play a role in potassium channel disorders, which are crucial for maintaining electrical activity and signaling in neurons and other cells, with mutations in *KCND3* and related genes causing intellectual disability [58]. Although a direct association of the *ZNF618* and *ZNF217* genes with fear is limited, these genes belong to the zinc finger proteins (ZNF) family, which has diverse functions [59]. Within the ZNF family, FEZ zinc finger 1 (*FEZF1*) [60], FEZ zinc finger 2 (*FEZF2*) [61], ZNF, and FOG family member 1 (*ZFPM1*) [62] contribute to the development and function of neural circuits, which are crucial for fear, anxiety, and behavioral regulation. Further studies are needed to fully understand the genetic evolution of fear-related traits in chickens, particularly within conserved genomic regions.

## 5. Conclusions

This study revealed that the six native Japanese breeds have moderate levels of genetic diversity and inbreeding compared to commercial breeds, highlighting the need for targeted conservation strategies and breeding management to enhance higher genetic diversity. Our population analysis identified four genetic groups that reflect fear-related behavioral tendencies in each group. Furthermore, seven SNPs were identified within the selection signatures. Five of these SNPs were candidate genetic variants for QTLs affecting fear behavior. These findings provide insights into genetic diversity and conserved genome segments valuable for breeding and conservation, with future studies needed on genomic regions linked to fear and behavior traits.

## Figures and Tables

**Table 1:** List of chicken breeds used in this study.

Breed	Abbreviation	Number of Animals		Source ^1^
		Male	Female	
Native Japanese chickens				
Ingie	IG	8		JABPC
Nagoya	NAG	10	3	NLBCH
Oh-Shamo	OSM	12		JABPC
Ryujin-Jidori	RYU	10		LPLESWP
Tosa-Jidori	TJI	11		JABPC
Tosa-Kukin	TKU	10		JABPC
Ukokkei	UK	6	1	JABPC
Foreign chickens				
White Plymouth Rock	WPR	12		NLBCH
Fayoumi inbred line	PNP	11		ARBC
White Leghorn G-line	WL-G	16		ARBC
White Leghorn T-line	WL-T	10		TCLM

^1^ JABPC, Japanese Avian Bioresource Project Research Center, Hiroshima University; NLBCH, National Livestock Breeding Center, Hyogo, Japan; LPLESWP, Laboratory of Poultry, Livestock Experimental Station, Wakayama Prefecture, Japan; ARBC, Avian Bioscience Center Nagoya University; TCLM, Tomaru Co., Ltd., Maebashi, Japan.

**Table 2:** Genetic diversity measurements across 11 populations of native Japanese and foreign chicken breeds.

Population	Seg. Sites ^1^	MAF ^2^	Private Allele ^3^	He ^4^	π (pi)	θ (Theta)	Tajima’s D
IG	11,364	0.53	0.10	0.10	0.09	0.10	−0.01 *
NAG	11,323	0.47	0.12	0.10	0.10	0.09	0.01 **
OSM	11,298	0.34	0.56	0.20	0.20	0.17	0.03 **
RYU	11,281	0.49	0.10	0.11	0.09	0.10	−0.01 **
TJI	11,321	0.38	0.27	0.13	0.13	0.14	−0.01 *
TKU	11,294	0.46	0.16	0.13	0.13	0.12	0.01 **
UK	11,216	0.37	0.44	0.14	0.18	0.18	0.00
PNP	11,384	0.86	0.01	0.01	0.04	0.04	−0.01 **
WL-G	11,191	0.40	0.09	0.06	0.07	0.08	−0.01 **
WL-T	11,100	0.41	0.22	0.17	0.13	0.12	0.01 **
WPR	11,291	0.34	0.50	0.19	0.20	0.16	0.03 **

^1^ Total number of segregating sites within population (Pop.); ^2^ minor allele frequency; ^3^ unique alleles found in one population and not present in other comparator populations; ^4^ heterozygosity; * and ** indicate significance of the chi-square test at *p*-values < 0.05 and <0.01, respectively.

**Table 3:** Overlapping SNPs between high ROH incidence and highest FST *p*-value.

Marker ID	CHR ^1^	Mb ^1^	ROH Incidence	FST	Nearest Gene ^1^	QTL ^2^
marker106395	13	16.65	53.78	0.89	Shroom Family Member 1 (*SHROOM1*)	Aggressiveness (17.4 Mb), feather pecking (17 Mb), receiving feather pecking (15.3)
marker113615	17	1.86	57.98	0.90	Zinc Finger Protein 618 (*ZNF618*)	
marker123266	20	12.31	40.00	0.89	Replication Termination Factor 2 Domain-Containing 1 (*RTFDC1*)	Tonic immobility (TI) duration (10.9–13.4)
marker123400	20	12.81	39.00	0.86	Zinc Finger Protein 217 (*ZNF217*)	
marker128107	24	3.58	57.14	0.84	*LOC107055055*	TI duration (1.8–3 Mb), TI attempts (3.3–4.4 Mb), feather pecking (3.3 Mb)
marker130448	26	3.41	59.66	0.89	Potassium Voltage-Gated Channel Subfamily D Member 3 (*KCND3*)	Aggressiveness (2.6 Mb)
marker130642	26	4.09	57.98	0.86	Nerve Growth Factor (*NGF*)	Feather pecking (3.7 Mb)

^1^ Location based on reference sequence GCF_016699485.2. ^2^ Retrieved from Animal QTLdb database [46].

## Data Availability

The RAD-seq data were deposited with the DNA Data Bank of Japan (DDJB) under accession numbers DRX438349–DRX438468. The data presented in this study are available in the Supplementary Materials.

## Supplementary Materials

The following supporting information can be downloaded at: https://www.mdpi.com/article/10.3390/ani14223341/s1, Table S1: Analysis of molecular variance (AMOVA); Table S2: Euclidean distances (lower diagonal) and Fst distances (upper diagonal) between 11 populations of chicken breeds and lines; Table S3: Runs of homozygosity per individual; Table S4: Run of homozygosity (ROH) segments identified across populations; Table S5: Results of OutFLANK analysis; Table S6: Incidence of ROH of SNPs across genome; Figure S1: Distribution of eigenvalues across principal coordinate (PCo) axes.
