# Supplementary figures and images for: Genetic Diversity, Runs of Homozygosity, and Selection Signatures in Native Japanese Chickens: Insights from Single-Nucleotide Polymorphisms
Source: Animals (Basel). 2024 Nov 20;14(22):3341. doi: 10.3390/ani14223341 (PMC11591434; doi:10.3390/ani14223341)

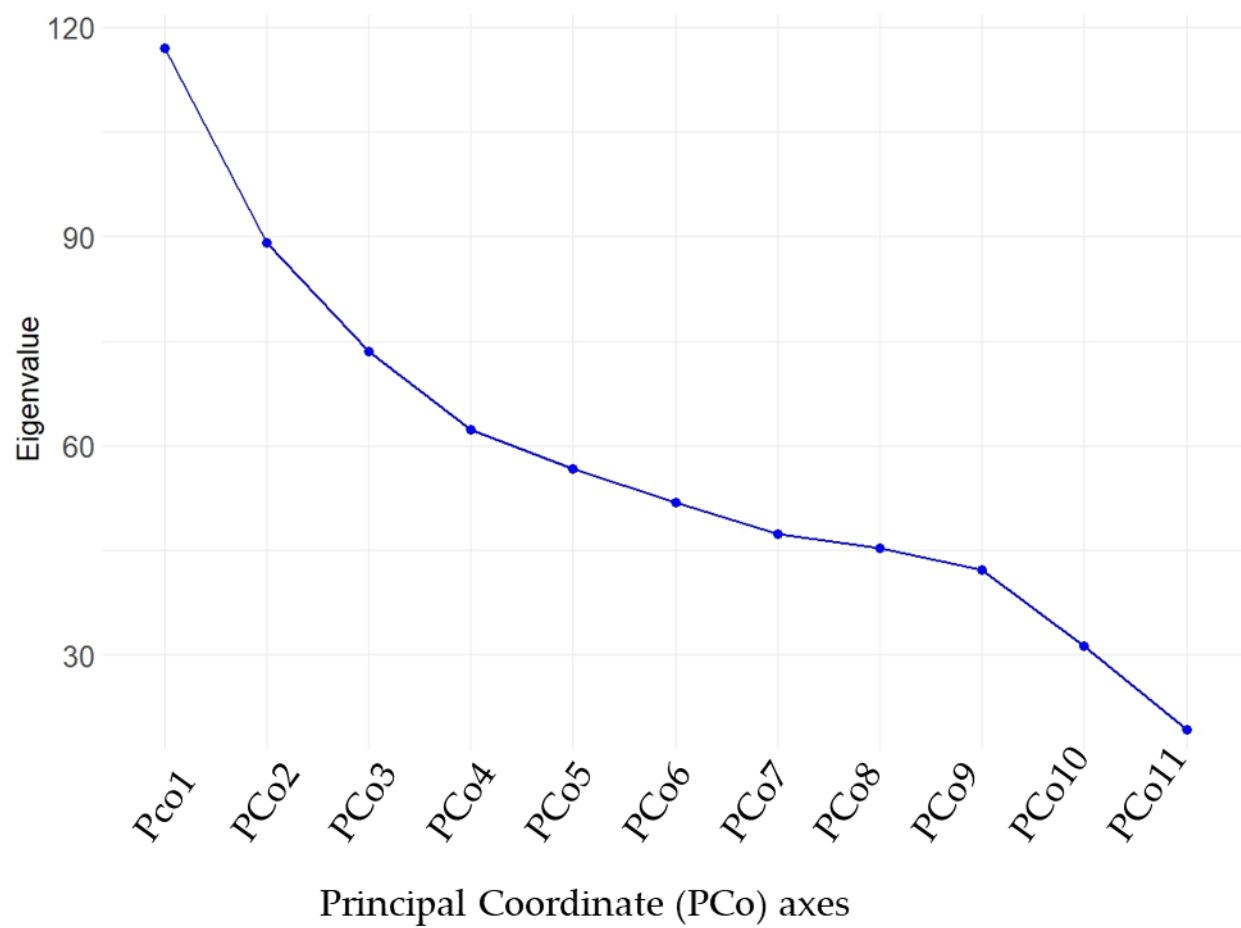

**Figure S1.** Distribution of eigenvalues across principal coordinate (PCo) axes

Supplement: Supplementary file 1 [file animals-14-03341-s001.zip › Figure S1.pdf]
